# Cross-validation of the factorial structure of the Neighborhood Environment Walkability Scale (NEWS) and its abbreviated form (NEWS-A)

**DOI:** 10.1186/1479-5868-6-32

**Published:** 2009-06-09

**Authors:** Ester Cerin, Terry L Conway, Brian E Saelens, Lawrence D Frank, James F Sallis

**Affiliations:** 1 USDA/ARS Children's Nutrition Research Center, Baylor College of Medicine, 1100 Bates Street, Houston, Texas 77030, USA; 2 Graduate School of Public Health, San Diego State University, San Diego, California, USA; 3 Seattle Children's Hospital Research Center, University of Seattle, UW-CHI NE 74th St, Box 354920, Seattle, Washington 98101, USA; 4 School of Community and Regional Planning, University of British Columbia, 231-1933 West Mall, Vancouver, BC V6T 1Z2, Canada; 5 Department of Psychology, San Diego State University, 3900 Fifth Avenue, San Diego, California 92103, USA

## Abstract

**Background:**

The Neighborhood Environment Walkability Scale (NEWS) and its abbreviated form (NEWS-A) assess perceived environmental attributes believed to influence physical activity. A multilevel confirmatory factor analysis (MCFA) conducted on a sample from Seattle, WA showed that, at the respondent level, the factor-analyzable items of the NEWS and NEWS-A measured 11 and 10 constructs of perceived neighborhood environment, respectively. At the census blockgroup (used by the US Census Bureau as a subunit of census tracts) level, the MCFA yielded five factors for both NEWS and NEWS-A. The aim of this study was to cross-validate the individual- and blockgroup-level measurement models of the NEWS and NEWS-A in a geographical location and population different from those used in the original validation study.

**Methods:**

A sample of 912 adults was recruited from 16 selected neighborhoods (116 census blockgroups) in the Baltimore, MD region. Neighborhoods were stratified according to their socio-economic status and transport-related walkability level measured using Geographic Information Systems. Participants self-completed the NEWS. MCFA was used to cross-validate the individual- and blockgroup-level measurement models of the NEWS and NEWS-A.

**Results:**

The data provided sufficient support for the factorial validity of the original individual-level measurement models, which consisted of 11 (NEWS) and 10 (NEWS-A) correlated factors. The original blockgroup-level measurement model of the NEWS and NEWS-A showed poor fit to the data and required substantial modifications. These included the combining of aspects of building aesthetics with safety from crime into one factor; the separation of natural aesthetics and building aesthetics into two factors; and for the NEWS-A, the separation of presence of sidewalks/walking routes from other infrastructure for walking.

**Conclusion:**

This study provided support for the generalizability of the individual-level measurement models of the NEWS and NEWS-A to different urban geographical locations in the USA. It is recommended that the NEWS and NEWS-A be scored according to their individual-level measurement models, which are relatively stable and correspond to constructs commonly used in the urban planning and transportation fields. However, prior to using these instruments in international and multi-cultural studies, further validation work across diverse non-English speaking countries and populations is needed.

## Background

Ecological models postulate that health behavior changes are a function of psychological, social, policy, and physical environmental factors [1,2]. Numerous authors and agencies have identified environmental and policy intervention as promising strategies for creating population-wide changes in physical activity and obesity [3-6]. Current evidence of a relationship between the built environment and physical activity is generally supportive but there are limitations [7]. An important limitation is that virtually all studies have been conducted in settings with restricted environmental variability. Restricted variability yields attenuated estimates of associations [8], so the magnitude of associations between environmental characteristics and physical activity is likely underestimated [9,10]. An accurate assessment of such associations requires greater environmental variability than any one country or region can offer. The International Physical Activity and the Environment Network (IPEN; ) has set out to support coordinated data collection in countries with diverse environments and populations. IPEN uses common study design and measurement to produce more reliable and valid effect size estimates.

The Neighborhood Environment Walkability Scale, (NEWS) [11] and its abbreviated form (NEWS-A) [12], are the measures of perceived neighborhood environment selected for the IPEN initiative. The NEWS and NEWS-A assess perceived environmental characteristics, stemming in part from the urban planning literature [13], believed to influence walking and other forms physical activity. Initial evidence for their criterion validity and reliability has been documented in four countries across four continents [11,14-17]. Except for the neighborhood characteristic of not many/any cul-de-sacs, test-retest reliability of the individual items of the NEWS and NEWS-A was moderate to high [14]. Significant associations were observed between the NEWS subscales and objective [18] as well as self-report measures of physical activity and walking [12,16,17]. Additionally, scores on the NEWS were strongly associated with corresponding objectively-measured constructs of neighborhood environments [11,16,19].

Two recent studies examined the measurement models of the original and Australian versions of the NEWS [12,16]. The measurement models described the relationships of the items to the theoretical constructs measured by the scales [20]. In other words, they identified groupings of items measuring distinct perceived neighborhood-environment constructs (e.g. environmental aesthetics, traffic hazards, and access to services).

To maximize the variability in environmental attributes, both studies adopted a stratified two-stage cluster sampling strategy whereby participants were recruited from specific areas (here, census blockgroups, the smallest geographical units for which census bureaus publish demographic data) selected according to their objectively-measured walkability and socio-economic status (SES; i.e. median income). Stratification by SES likely enhanced the representativeness of the sample because, otherwise, low SES respondents might have been underrepresented [21]. Two distinct measurement models of the NEWS and NEWS-A, one for each level of variation in the data, were examined to address violations of the statistical assumption of independence of observations resulting from the adopted sampling strategy [20]. Thus, measurement models were defined at the individual (based on within-census blockgroup variations in the responses to the items) and blockgroup levels (based on the between-census blockgroup variations) [12,16].

The individual-level measurement models were based on differences in responses between study participants living in the same blockgroups and described the way perceived environmental attributes (represented by the NEWS items) covaried within census blockgroups. The differences in responses may have resulted from actual environmental differences within a blockgroup (e.g. differences in traffic load or aesthetics across locations within a blockgroup), response biases (e.g. tendency to provide extreme ratings), and/or perceptual biases (e.g. anxious respondents' tendency to overestimate the risk of crime in their neighborhood) [12]. In contrast, the blockgroup-level measurement models were based on the blockgroup average ratings of the items and indicated how perceived environmental attributes covaried between blockgroups. These models likely reflected the way environmental attributes clustered objectively across blockgroups. In fact, the average rating of a blockgroup characteristic can be considered a relatively reliable and valid indicator of the objective environment. This is because response and perceptual biases are likely to be random effects that, by definition, cancel out when summed across respondents and, hence, have no impact on the average rating for a blockgroup. Importantly, blockgroup-level factors were found to be strongly correlated with corresponding objective measures [16].

The authors recommended scoring the NEWS according to the individual-level measurement model for three main reasons: (1) the individual-level factors more accurately represented constructs commonly used in the urban planning and transportation fields [13]; (2) they likely indicate how perceptions of environmental attributes group together into factors, while blockgroup-level factors likely represent patterns of associations between objective environmental attributes; (3) they are likely to be more generalizable across locations and populations than are blockgroup-level factors [12].

The measurement model of the Australian NEWS mostly resembled that of the original version, but differed in significant ways [16]. For instance, while traffic-related items formed a unique individual-level latent factor in the original NEWS tested in some USA cities, in the Australian version they split into two weakly correlated factors – namely, traffic safety and traffic hazards. Although dissimilarities in factorial structures were partly attributed to substantive item-content differences between the two versions of the NEWS [16], they also raise concerns about the reliability and generalizability of the original measurement model to different geographical and cultural settings. Hence, it was necessary to cross-validate the original NEWS and NEWS-A in a geographical location and population different from those used in the original validation study (i.e. Seattle, Washington region). Such information is important for establishing common, valid scoring protocols, which in turn can provide a more accurate estimation of a dose-response relationship of the perceived built environment with physical activity and obesity. Thus, the current paper reports the individual- and blockgroup-level factor structures of the NEWS and NEWS-A tested in the Baltimore, Maryland – Washington, DC region, which is a demographically- and environmentally-dissimilar city to Seattle, WA.

Specifically, according to the 2003 American Community Survey, Seattle was the most educated larger city in the USA, with 52% of residents aged 25 and over having attained at least a bachelor's degree [22]. In contrast, the percentage of highly educated residents in Baltimore was 35.6. The 2000 median household income in Baltimore was approximately $32,500, while in Seattle it was $49,500. Seattle had 70.5% of White and only 7.8% of African American residents, while the percentage of African Americans in Baltimore was 63.8, and that of Whites 31.4. Baltimore is located on the East coast of the United States, while Seattle is located on the West coast. Baltimore and Seattle are similar in size, terrain, urban layout (grid pattern), and are both considered "cities of neighborhoods". However, with its climate and geographical location, Seattle provides more ample access to a variety of outdoor activities. Also, Seattle has higher population density, more traffic congestion problems, but lower crime rates than Baltimore [22,23].

We hypothesized that the individual-level factor structures of the NEWS and NEWS-A, derived from the original validation sample (Seattle, Washington, USA), would show a sufficient level of fit to the data from the cross-validation sample (Baltimore, MD – Washington, DC, USA) due to them being in part a function of psychological principles that apply across diverse subgroups. We also hypothesized that the original blockgroup-level factor structures of the NEWS and NEWS-A would show poorer fit to the data from the cross-validation sample than their individual-level counterparts due to them reflecting patterns of associations between objective environmental factors, which likely vary across geographical locations.

## Methods

### Participants

This study used cross-sectional survey data from the Neighborhood Quality of Life Study (NQLS) conducted in the Baltimore, MD – Washington, DC region. Neighborhoods, defined as clusters of blockgroups, were selected to vary in walkability characteristics and socio-economic status (SES). Median household income was used to define blockgroup SES, while data within a Geographic Information System (GIS) on residential density, street connectivity, land-use mix, and retail floor area ratio were used to operationalize blockgroup walkability [24]. Blockgroups were deciled based on their walkability levels and the lowest (deciles 1–3) and highest (deciles 7–10) were selected for potential recruitment. Income was also deciled within these selected blockgroups and blockgroups within deciles 2–4 and 7–9 were selected for potential recruitment. The end result was the selection of participants from 16 neighborhoods (116 census blockgroups) whose blockgroups met the specified walkability and income criterion [24].

Households within selected blockgroups were identified by a marketing firm, sent an invitation letter, and then called within 2 weeks of the expected receipt of this letter. An adult in the household was asked about interest and study eligibility. A sample of 912 (19%; 912 participants/4,816 eligible people contacted) English-speaking adults, aged 20–65, able to walk without assistance, and living in private dwellings, was recruited. Participants' socio-demographic characteristics are shown in Table 1.

**Table 1:** Socio-demographic characteristics of the sample (N = 912)

Characteristic	Estimate	Characteristic	Estimate
**Gender, %**		**Age, mean (SD), y**	46.6 (10.7)
Female	52.3	Missing values, %	0.1
Missing values	0.0	**Marital status, %**	
**Ethnicity, %**		Married	54.4
Caucasian	61.9	Widowed/divorced/separated	18.9
African-American	27.2	Single/never married	20.4
Asian-American	3.0	Living with partner	5.7
Pacific Islander	0.1	Missing values	0.6
Amer. Indian/Alaskan Native	0.4	**Children in household, %**	
Hispanic	3.2	Yes	39.6
Other	3.1	Missing values	0.5
Missing values	1.1	**Annual household income, %**	
**Educational attainment, %**		< $19,500	5.2
Some high school or less	2.1	$19,500 – $39,500	12.9
Completed high school	7.3	$39,500 – $59,500	17.8
Some college	22.9	$59,500 – $79,500	15.9
Completed college	30.5	$79,500 – $99,500	13.8
Completed graduate degree	36.7	> $99,500	27.3
Missing values	0.5	Missing values	7.1

### Measures

#### Socio-demographic characteristics

Participants self-reported gender, age, educational attainment, annual household income, marital status, and number of children (≤ 18 years old) in the household.

#### Neighborhood Environment Walkability Scale (NEWS and NEWS-A)

The NEWS and NEWS-A (abbreviated version) consist of 67 and 54 items, respectively [12]. These are grouped into eight multi-item subscales (representing distinct constructs or latent factors) including perceived residential density; proximity to nonresidential land uses (land use mix – diversity); ease of access to nonresidential uses (land use mix – access); street connectivity; infrastructure for walking and cycling; aesthetics; traffic safety; and safety from crime. The first two subscales are not factor-analyzable and, hence, represent constructs rather than latent factors. Five single-item subscales (four in the NEWS-A) assess perceived major physical barriers to walking; hilly streets; difficult car parking in shopping areas; absence of cul-de-sacs; and presence of people being active in the neighborhood (not included in the NEWS-A). All subscales, with the exception of residential density and land use mix – diversity, are rated on a 4-point Likert scale. Residential density items are rated on a 5-point scale, and ratings are weighted relative to the average residential density that a specific item represents [11]. The weighted ratings are summed to create a perceived residential density score. Land use mix – diversity is assessed by the perceived walking proximity from home to various types of destinations, with responses ranging from 1- to 5-minute walking distance (coded as 5) to >30-min walking distance (coded as 1).

### Procedure

One interested and eligible adult per household was sent the consent form and, upon its return, was sent questionnaires with instructions and postage paid return envelope or was sent a link via e-mail to complete the survey online. The study was approved by the ethics committee of participating research institutions.

### Data Analyses

Multilevel confirmatory factor analysis (MCFA) was employed to estimate the individual- and blockgroup-level measurement models of the factor-analyzable items (all but residential density and land use mix – diversity items; i.e. six subscales in total) of the NEWS and NEWS-A. The analyses were multilevel because the study adopted a two-stage cluster sampling design and substantial intraclass correlation coefficients (ICCs; denoting the proportion of total item variance due to differences between blockgroups) were observed at the blockgroup level. The average ICC was .22 (range: 0.02 to 0.42).

MCFA was conducted using Bentler and Liang's Maximum Likelihood Estimation (MLE) method, applicable to multilevel samples with clusters (e.g. blockgroups) varying in size [25]. Empirically-derived a priori two-level measurement models of the NEWS and NEWS-A were tested [12]. The a priori models consisted of six individual-level correlated factors (see Measures section) and five blockgroup-level correlated factors [12]. Additionally, the model of the NEWS had five, and that of the NEWS-A four, single items. A well-fitting individual-level model would suggest that the pattern of correlations between individual differences in perceived attributes of the neighborhood environment observed in a sample of Seattle residents is generalizable to residents of the Baltimore – Washington region. A well-fitting blockgroup-level model would indicate that the pattern of correlations between average perceived attributes of the neighborhood environment observed in a sample of Seattle neighborhoods is generalizable to the selected neighborhoods from the Baltimore-Washington region.

Re-specification of the a priori models was based on Jöreskog and Sörbom's iterative model-generating approach [26], whereby inadequate fit of the data to the model is followed by re-specification of the model so to achieve a statistically acceptable fit and a theoretically meaningful interpretation of the data. Model re-specification was guided by the analysis of standardized factor loadings, standardized residual covariances, univariate Langrage multiplier tests, and Wald tests, and theoretical issues [26]. Factor loadings equal or greater |.30| were considered to be significant [27].

The measures of model fit included the Bentler-Liang likelihood ratio (LR) statistic, the Goodness-of-Fit Index (GFI), the Root Mean Square Error of Approximation (RMSEA), the Non-Normed Fit Index (NNFI), the Comparative Fit Index (CFI), and the Standardized Root Mean Squared Residual (SRMS) [20,28]. We also used the Aikake Information Criterion (AIC), including a penalty for model complexity and allowing the comparison of non-nested models [20]. The following cut-off values of acceptable model fit were adopted: >.95 for CFI, NFI, and GFI; <.08 for SRMR; and <.06 for RMSEA [29]. Analyses were performed using EQS 6.1 (Multivariate Software Inc., Encino, CA, 2004).

## Results

All but two items had acceptable values of univariate skewness (< 2.0) and kurtosis (< 7.0) for the use of maximum likelihood estimation [30]. The average skewness and kurtosis of items were 0.32 and 0.57, respectively. The items 'There are canyons/hillsides in my neighborhood' (item 7) and 'The crime rate in my neighborhood makes it unsafe to go on walks during the day' (item 36) had skewness 2.67 and 2.26, respectively. Although higher than recommended, these two values are likely to yield ignorable estimation biases [30].

Table 2 shows the model fit indices for the a priori and re-specified measurement models of the NEWS and NEWS-A. The application of the present data to the a priori measurement model of the NEWS, based on its empirically-derived model [12], met three (GFI≥0.95; RMSEA<0.06; SRMR < 0.08) out of five goodness-of-fit criteria (Table 2). The a priori measurement model of the abbreviated version of the NEWS (NEWS-A) showed an even better fit, with four (GFI≥0.95, RMSEA<0.06, NNFI≥0.95, and CFI≥0.95) out of five indices meeting the adopted cut-off values.

**Table 2:** Results of the multilevel CFAs of the NEWS and NEWS-A on the cross-validation sample: Model fit indices

Model	χ^2^	df	GFI	RMSEA (90% CI)	SRMR	NNFI	CFI	AIC
**a) NEWS**								
Model 1: A priori	2881	1135	1.00	.038 (.035 – .041)	.066	.91	.92	609
Model 2: Re-specified	2801	1135	1.00	.036 (.033 – .040)	.070	.92	.92	531
								
**b) NEWS-A**								
Model 1a: A priori	1099	445	1.00	.030 (.024 – .035)	.113	.95	.96	211
Model 2a: Re-specified	1066	445	1.00	.026 (.020 – .032)	.076	.96	.97	178

### Individual-level measurement models

The individual-level models of the NEWS and NEWS-A yielded acceptable standardized factor loadings for all but one item ('Sidewalks are separated from the road/traffic in my neighborhood by parked cars'; Table 3). As this item showed the highest standardized loadings (i.e. .25 for the NEWS and .26 for the NEWS-A) with the factor it was supposed to measure ('Infrastructure and safety for walking/cycling') followed by 'Land use mix – access' (.21 for the NEWS and .18 for the NEWS-A), no modifications were made to the a priori individual-level model of the NEWS and NEWS-A. The correlations between individual-level factors of the NEWS and NEWS-A are reported in Tables 4 and 5, respectively (above the diagonals). All items' standardized loadings were significant at the 0.001 level.

**Table 3:** Standardized factor loadings and uniquenesses for final re-specified individual-level and neighborhood-level measurement models of the NEWS and NEWS-A (in brackets).

		Individual-level			Blockgroup-level		
	Item	SL	SU	LF	SL	SU	LF

1	Shopping at local stores	.66 (-)	.56(-)	IL1 (-)	.75 (-)	.44 (-)	BL1 (-)
2	Stores within easy walking distance	.85 (.63)	.28 (.61)	IL1 (IL1_A_)	.95 (.95)	.09 (.09)	BL1 (BL1_A_)
3	Parking difficult	Single item			.46 (.40)	.79 (.84)	BL1 (BL1_A_)
4	Many places within walking distance	.68 (.91)	.54 (.17)	IL1 (IL1_A_)	.90 (.90)	.19 (.19)	BL1 (BL1_A_)
5	Easy to walk to a transit stop	.31 (.30)	.90 (.90)	IL1 (IL1_A_)	.83 (.83)	.31 (.31)	BL1 (BL1_A_)
6	Hilly streets	Single item			.99 (.70)	.02 (.51)	BL2 (BL2_A_)
7	Major barriers to walking	Single item			.34 (.49)	.89 (.76)	BL2 (BL2_A_)
8	Few cul-de-sacs	Single item			.60 (.56)	.64 (.69)	BL1 (BL1_A_)
9	Short distance between intersections	.34 (.37)	.88 (.86)	IL2 (IL2_A_)	.86 (.88)	.26 (.22)	BL1 (BL1_A_)
10	Four-way intersections	.57 (-)	.67 (-)	IL2 (-)	.88 (-)	.23 (-)	BL1 (-)
11	Many alternative routes	.49 (.43)	.76 (.82)	IL2 (IL2_A_)	.92 (.92)	.15 (.15)	BL1 (BL1_A_)
12	Sidewalks	.50 (.41)	.75 (.83)	IL3 (IL3_A_)	.98 (.74)	.04 (.45)	BL3 (BL1_A_)
13	Well-maintained sidewalks	.58 (-)	.66 (-)	IL3 (-)	.60 (-)	.64 (-)	BL3 (-)
14	Bicycle or pedestrian trails	.41 (-)	.83 (-)	IL3 (-)	.38 (-)	.86 (-)	BL4 (-)
15	Cars dividing sidewalk and traffic	.25 (.26)	.94 (.93)	IL3 (IL3_A_)	.81 (.84)	.35 (.29)	BL1 (BL1_A_)
16	Grass/dirt dividing sidewalk and traffic	.30 (.31)	.90 (.90)	IL3 (IL3_A_)	.90 (.73)	.19 (.47)	BL4 (BL3_A_)
17	Safe to ride	.64 (-)	.56 (-)	IL3 (-)	.84 (-)	.29 (-)	BL5 (-)
18	Trees	.43 (.42)	.81 (.82)	IL4 (IL4_A_)	.85 (.99)	.27 (.02)	BL4 (BL3_A_)
19	Trees give shade	.41 (-)	.83 (-)	IL4 (-)	.64 (-)	.59 (-)	BL4 (-)
20	Many interesting things to look at	.72 (.77)	.48 (.41)	IL4 (IL4_A_)	.55 (.64)	.69 (.60)	BL5 (BL4_A_)
21	No litter	.51 (-)	.74 (-)	IL4 (-)	.92 (-)	.16 (-)	BL5 (-)
22	Many attractive natural sights	.72 (.71)	.49 (.50)	IL4 (IL4_A_)	.91 (.86)	.18 (.26)	BL5 (BL4_A_)
23	Attractive buildings/homes	.71 (.69)	.50 (.52)	IL4 (IL4_A_)	.63 (.71)	.60 (.50)	BL5 (BL4_A_)
24	Heavy traffic along the street	.73 (-)	.47 (-)	IL5 (-)	-.71 (-)	.50 (-)	BL6 (-)
25	Heavy traffic along nearby streets	.72 (.66)	.48 (.57)	IL5 (IL5_A_)	-.64 (-.56)	.59 (.69)	BL6 (BL5_A_)
26	Slow traffic speed on the street	-.50 (-)	.75 (-)	IL5 (-)	.87 (-)	.25 (-)	BL6 (-)
27	Slow traffic speed on nearby streets	-.48 (-.55)	.77 (.69)	IL5 (IL5_A_)	.75 (.71)	.44 (.50)	BL6 (BL5_A_)
28	Speeding drivers	.42 (.42)	.82 (.82)	IL5 (IL5_A_)	-.45 (-.60)	.80 (.64)	BL6 (BL5_A_)
29	Street lights	.49 (.60)	.76 (.64)	IL3 (IL3_A_)	.68 (.71)	.54 (.50)	BL1 (BL1_A_)
30	Walkers and bikers easily seen	.49 (.61)	.76 (.63)	IL3 (IL3_A_)	.81 (.87)	.34 (.25)	BL6 (BL5_A_)
31	Crosswalks and pedestrian signals	.34 (.30)	.88 (.90)	IL3 (IL3_A_)	.90 (.82)	.19 (.32)	BL1 (BL1_A_)
32	Crosswalks help walkers feel safe	.49 (-)	.76 (-)	IL3 (-)	.89 (-)	.21 (-)	BL1 (-)
33	Exhaust fumes	.50 (-)	.75 (-)	IL5 (-)	-.84 (-)	.30 (-)	BL5 (-)
34	Seeing/speaking to other people	Single item (NEWS only)			.61 (-)	.63 (-)	BL6 (-)
35	High crime rate	.76 (.75)	.43 (.44)	IL6 (IL6_A_)	-.98 (-.99)	.04 (.02)	BL5 (BL4_A_)
36	Unsafe to walk during the day	.55 (.52)	.70 (.73)	IL6 (IL6_A_)	-.92 (-.95)	.17 (.10)	BL5 (BL4_A_)
37	Unsafe to walk at night	.80 (.81)	.35 (.34)	IL6 (IL6_A_)	-.97 (-.98)	.06 (.03)	BL5 (BL4_A_)
38	Safe for children to walk alone	-.39 (-)	.85 (-)	IL6 (-)	.78 (-)	.39 (-)	BL5 (-)

(-) = not applicable. SL = standardized loadings; SU = standardized uniqueness; LF= latent factor. *Latent individual-level *factors: IL1 and IL1_A _= Land use mix – access; IL2 and IL2_A _= Street connectivity; IL3 = Infrastructure and safety for walking/cycling; IL3_A _= Infrastructure and safety for walking; IL4 and IL4_A _= Aesthetics; IL5 and IL5_A _= Traffic hazards; IL6 and IL6_A _= Crime. *Latent blockgroup-level *factors: BL1 and BL1_A _= Land use mix access and infrastructure for walking; BL2 and BL2_A _= Physical obstacles to walking; BL3 = Sidewalks; BL4 and BL3_A _= Green areas; BL5 and BL4_A _= Aesthetics and safety from crime; BL6 and BL5_A _= Traffic safety and presence of pedestrians. Autocorrelated error terms were modeled for items 18 and 19 (r = 0.55; t = 14.4; *P *< 0.001), and 31 and 32 (r = 0.59; t = 15.6; *P *< 0.001).

**Table 4:** Correlations between individual-level latent factors of the NEWS (above the diagonal) and NEWS-A (below the diagonal).

NEWS-A factors		IL2	IL3	IL4	IL5	IL6	NEWS factors
Land use mix – access (IL1_A_)		.29	.21	.19	< .10^a^	< .10^a^	Land use mix – access (IL1)
Street connectivity (IL2_A_)	.45		.32	.23	-.10	< .10^a^	Street connectivity (IL2)
Infrastructure & safety for walking (IL3_A_)	.17	.41		.43	-.59	-.40	Infrastructure & safety for walking/cycling (IL3)
Aesthetics (IL4_A_)	.29	.31	.30		-.42	-.31	Aesthetics (IL4)
Traffic hazards (IL5_A_)	< .10^a^	-.34	-.46	-.31		.51	Traffic hazards (IL5)
Crime (IL6_A_)	< .10^a^	< .10^a^	-.37	-.21	.46		Crime (IL6)

	IL1_A_	IL2_A_	IL3_A_	IL4_A_	IL5_A_		

^a ^constrained to zero in the final model as correlation coefficients smaller than |.10|. The subscript A stands for NEWS-A.

**Table 5:** Correlations between blockgroup-level latent factors of the NEWS (above the diagonal) and NEWS-A (below the diagonal).

NEWS-A factors		BL2	BL3	BL4	BL5	BL6	NEWS factors
Land use mix – access and infrastructure for walking (BL1_A_)		-.29	.66	< .10^a^	-.51	.19	Land use mix – access and infrastructure for walking (BL1)
Physical obstacles to walking (BL2_A_)	-.54		< .10^a^	< .10^a^	< .10^a^	- .14	Physical obstacles to walking (BL2)
Green areas (BL3_A_)	< .10^a^	< .10^a^		.41	-.46	.26	Sidewalks (BL3)
Aesthetics and safety from crime (BL4_A_)	-.46	< .10^a^	.47		.42	.36	Green areas (BL4)
Traffic safety and presence of people (BL5_A_)	.52	-.45	.57	.18		.39	Aesthetics and safety from crime (BL5)
	BL1_A_	BL2_A_	BL3_A_	BL4_A_			Traffic safety and presence of people (BL6)

^a ^constrained to zero in the final model as correlation coefficients smaller than |.10|. The subscript A stands for NEWS-A.

### Blockgroup-level measurement models

Although the values of the fit indices were similar to those obtained for the final model for the NEWS in the prior MCFA [12], an analysis of standardized item loadings of the blockgroup-level measurement model revealed lower than acceptable loadings (i.e. <|.30|) for eight items (# 6, 7, 13, 16, 19, 26, 28, and 34) (data not shown).

Based on analyses of indices of poor model fit and theoretical considerations, the blockgroup-level model of the NEWS was modified. While the a priori model included five correlated factors (i.e. land use mix access and infrastructure for walking; physical obstacles for walking/cycling; aesthetics and friendliness; traffic hazards; and crime), the data in the present study supported the existence of six correlated factors (Table 3). Items describing natural physical obstacles to walking (items 6 and 7) formed a factor ('Physical obstacles to walking') separate from items related to lack of facilities for cycling. Items 12 and 13, describing sidewalks, combined into a unique factor, while, in the prior MCFA [12], they loaded on the latent factors 'Obstacles to walking/cycling' and 'Land use mix – access and infrastructure for walking'. Contrary to the a priori model definition, items related to greenness ('Green areas') formed a factor separate from other aesthetic aspects of the environment (e.g. attractive buildings and cleanliness). Item 17 ('It is safe to ride a bike in or near my neighborhood') was related to environmental aesthetics, as were items describing level of crime safety in the neighborhood ('Aesthetics and crime safety'). Similar to the a priori model, all traffic-related items loaded onto a single factor ('Traffic safety and presence of pedestrian'). While the a priori model defined presence of pedestrians as a characteristic associated with aesthetics and traffic safety, the data supported associations with traffic safety only. Four goodness-of-fit indices (χ^2^, RMSEA, NNFI, and AIC) indicated a better fit of the re-specified than the a priori model to the data (Table 2).

Similar to the model of the NEWS, an analysis of standardized factor loadings indicated a certain degree of misfit at the blockgroup-level of the NEWS-A model, whereby two items (16 and 25) had unacceptably low loadings (-.10 and -.21). An analysis of indices of poor model fit led to several modifications of the blockgroup-level model. The re-specified model showed excellent fit, with all indices meeting the goodness-of-fit criteria (Table 2). Both blockgroup-level a priori and re-specified models consisted of five correlated factors including 'Land use mix – access and infrastructure for walking' and 'Physical obstacles to walking'. However, while the a priori blockgroup-level model included the factors 'Aesthetics', 'Traffic hazards', and 'Safety from crime', the re-specified model included 'Green areas', 'Aesthetics and safety from crime', and 'Traffic safety and presence of pedestrians'. The correlations between blockgroup-level factors of the NEWS and NEWS-A are shown in Tables 4 and 5, respectively (below the diagonals). All items' standardized loadings were significant at the 0.001 level.

## Discussion

The main aim of this study was to cross-validate the factorial structure of the NEWS [11,12] and NEWS-A [12] by comparing prior analyses in one USA metropolitan area to another USA metropolitan area. The overall goodness of fit of the a priori model of the NEWS was similar to that reported in the original validation study, while that for the NEWS-A was slightly lower [12]. Support was found for the validity of the current individual-level measurement models consisting of six correlated factors (land use mix – access; street connectivity; infrastructure and safety for walking; aesthetics; traffic hazards; and crime) and five (NEWS) or four (NEWS-A) single items. In contrast, the blockgroup-level models of the NEWS and NEWS-A showed a poor fit to the data. Re-specification of the blockgroup-level models resulted in goodness of fit level comparable to those from the original validation study for both NEWS and NEWS-A [12]. The individual- and blockgroup-level measurement models are discussed in detail below.

### Individual-level measurement models

As noted earlier, the a priori individual-level measurement model fitted the data well. Only the item 'Sidewalks are separated from road/traffic in my neighborhood by parked cars' insufficiently, yet maximally, loaded on the factor it was supposed to represent ('Infrastructure and safety for walking/cycling'). It is noteworthy that the same item had a relatively low loading in the original validation study of the NEWS (0.38) [12] and did not sufficiently load on any factors in a validation study of the Australian version of the NEWS [16]. In the present study, this item also tended to correlate with the factor representing access to facilities, while in the Australian sample it showed a strong positive association with walking for transportation at the blockgroup level.

It appears that the separation of sidewalks from traffic by parked cars may be indicative of access to destinations (i.e. parked cars at nearby services) as well as infrastructure for walking (i.e. sidewalks). However, this environmental characteristic is less likely to be associated with pedestrian safety. Cars parked along sidewalks are a sign of local motorized traffic that may pose problems to pedestrians wishing to cross a road, especially in the absence of crosswalks. This may explain why this particular environmental characteristic weakly or inconsistently loaded on the infrastructure and safety dimension. At present, before more information on the factorial validity of the NEWS across various geographical locations is gathered, it is suggested the item 'Sidewalks are separated from road/traffic in my neighborhood by parked cars' be considered part of the infrastructure and safety factor, particularly given that both item and factor (excluding and including the item) were found to be significantly positively related to weekly minutes of walking for transport [12,16] and, marginally, positively related to walking for recreation [16].

### Blockgroup-level measurement models

In this study, the a priori blockgroup-level models of the NEWS and NEWS-A did not show a sufficient level of fit to the data. The main differences between the poorly-fitting a priori and the well-fitting re-respecified blockgroup level models were (1) the separation of natural (i.e. trees and natural sights) from building aesthetics; (2) a stronger association between building aesthetics and crime; (3) and the separation of sidewalks from other infrastructure for walking (i.e. crosswalks, grass strips, and street connectivity).

As noted earlier, blockgroup-level factors were expected to be less stable and generalizable across locations than individual-level factors for two main reasons. First, blockgroup-level associations depend on the criteria for the selection of study areas. Second, blockgroup-level factors are more likely to represent patterns of associations between objective environmental factors, which can substantially vary across geographical locations. For example, high levels of household density and access to services may, in certain urban environments, be associated with lower socio-economic status and higher crime [31], while in others this pattern may be related to higher socio-economic status and higher levels of aesthetics [32,33]. Also, a comparison of the results from the present study with those from the original validation study of the NEWS [12] suggests a stronger association between greenery and blockgroup socio-economic status (represented by higher levels of building aesthetics), but a weaker association between aesthetics and crime, in selected neighborhoods of Seattle than Baltimore regions.

In contrast, individual perceptions of environmental characteristics are likely to be in part a function of psychological principles that apply across diverse subgroups. Experimental studies indicate that the evaluation of concepts believed to be related substantially influence perceptions of these concepts [34]. People tend to exaggerate differences among items/attributes that fall into different conceptual categories, and minimize differences among items/attributes that fall into the same category [35]. Such a mechanism would explain the respondents' tendency to group environmental characteristics into meaningful, distinct concepts (e.g. access to services, street connectivity, and crime) common to their culture and language irrespective of their place of residence. Hence, we recommend using the individual-level measurement models of the NEWS and NEWS-A in both single- and multi-site studies.

### Limitations and future research

The relatively low response rate is one of the main limitations of the study. This is likely due in part to the extensive measurement protocol, including surveys and accelerometer monitoring on two occasions. Because recruitment rates did not differ by walkability/income quadrants, differential selection bias seems unlikely. However, the fact that similar individual-level measurement models were observed across three geographical locations (Baltimore, Seattle, and Adelaide) assuages concerns about sample bias effects. Another limitation pertains to the adopted sampling design that, while facilitating the recruitment of a socio-economically balanced sample, precluded the derivation of blockgroup-level measurement models of the NEWS and NEWS-A representative of the geographical locations. It is possible that a random sample of blockgroups might have resulted in greater similarities between the individual- and blockgroup-level measurement models and higher levels of generalizability of the blockgroup-level measurement model across geographical locations. This is because the procedure for the selection of blockgroups adopted in the three validation studies of the NEWS might have artificially inflated the blockgroup-level correlation between certain environmental features (street connectivity and land use mix – access).

It is important to note that all three validation studies of the NEWS were conducted in the USA and Australia, two countries with similar cultures and language, as well as a preponderance of low-density land uses. This may have in part contributed to the observed similarities among the individual-level measurement models. It is yet to be seen whether the current measurement model of the NEWS can be replicated in populations outside Australia and the USA, although we hypothesize that it is likely to be sufficiently generalizable to other countries and cultures. The reason for this is that most the items of the NEWS depict tangible, physical neighborhood attributes, with the exception of safety-related items. We believe that the interpretation of items dealing with physical attributes is less likely to differ across cultures than is that of items gauging socio-cultural and psychological concepts. Yet, empirical confirmation for our hypothesis is needed.

A limitation on generalizability is likely to be incomplete assessment by the NEWS and NEWS-A of environmental attributes found in other geographic locations. Different forms of mixed use, different pedestrian and bicycling infrastructure, and different public transit access and facilities in other countries should be reflected in modified versions of the NEWS and NEWS-A. Efforts to make such modifications are currently ongoing.

## Conclusion

This study provided further support for the factorial validity of the NEWS and its abbreviated version. At present, it is recommended that the NEWS and NEWS-A be scored according to the individual-level model comprising eight multi-item subscales and five (for the NEWS) or four (for the NEWS-A) single-item subscales. As these subscales are clearly related to constructs used in urban planning and transportation, findings based on these subscales can inform policies and interventions that may improve the activity-friendliness of a neighborhood. Given that all factorial-validation studies of the NEWS and NEWS-A were conducted on English-speaking populations, before they can be 'comfortably' used in global or multi-cultural studies further validation work across diverse populations is needed.

## Competing interests

The authors declare that they have no competing interests.

## Authors' contributions

EC analyzed the data, conceptualized and wrote all drafts of the manuscript. BES, LDF and JFS designed, organized and conducted the Neighborhood Quality of Life Study (NQLS). TLC contributed to the coordination of NQLS, data management, and preliminary data analyses for this paper. TLC, BES, LDF, and JFS critically reviewed and edited all drafts of the manuscript. All authors approved the final version of the manuscript.
